# Natural Source-Based Graphene as Sensitising Agents for Air Quality Monitoring

**DOI:** 10.1038/s41598-019-40433-9

**Published:** 2019-03-07

**Authors:** R. Parvizi, S. Azad, K. Dashtian, M. Ghaedi, H. Heidari

**Affiliations:** 1 Department of Physics, College of Sciences, Yasouj University, Yasouj, 75914-353 Iran; 2 Chemistry Department, Yasouj University, Yasouj, 75914-353 Iran; 3 School of Engineering, University of Glasgow, Glasgow, G12 8QQ United Kingdom

## Abstract

Natural carbon powder has been used as a precursor to prepare two main types of sensitising agents of nitrogen-doped carbon nanoparticles (N-CNPs) and nitrogen-doped graphene quantum dots coupled to nanosheets (N-GQDs-NSs) by using simple treatments of chemical oxidation and centrifugation separation. Characterization based on FTIR, XPS, XRD, Raman spectroscopy, FE-SEM, HR-TEM, AFM, UV-Vis and FL, revealed successful doping carbon nanoparticle with nitrogen with an average plane dimension of 50 nm and relatively smooth surface. The versatility of the prepared samples as sensitising agents was developed and established by exploiting its ability for detection of volatile organic compounds via simple optical fibre based sensing configuration. The comparative experimental studies on the proposed sensor performance indicate fast response achieved at a few tens of seconds and excellent repeatability in exposure to the methanol vapour. The low limit of detection of 4.3, 4.9 and 10.5 ppm was obtained in exposure to the methanol, ethanol and propanol vapours, respectively, in the atmosphere condition. This study gives insights into the chemical/physical mechanism of an enhanced economic optical fibre based gas sensor and illustrates it for diverse sensing applications, especially for chemical vapour remote detection and future air quality monitoring.

## Introduction

In recent years, volatile organic compounds (VOCs) composed of carbon, metals, inorganic chemicals and organic hydrocarbons are primary sources of air pollutants. VOCs are efficiently emitted from the automotive and bio-fuel industries^1^, paints, building and furnishing materials^2^. In a certain amount, VOCs are believed to have short-term adverse health effects as contributing to such symptoms: allergies, loss of concentration, drying and irritation of mucous membrane of nose, throat and eyes^3^, and long-term fatal effects on human health, including the potential cause of cancer, typically as lung cancer^4^. Accordingly, the design of a portable sensor able to determine the VOCs concentration in each composition in a various environment is critical. Therefore, their quantification in gas phase based on an inexpensive sensor is of great commercial significance and favour with direct applications in other diverse fields of chemical industries, electrical productions, the environmental monitoring, foods productions^5^ and forensics (testing of alcohol consumption in humans)^6,7^. The well-known techniques for VOC detection classified as photo-ionization detectors^8^, gas chromatography^9^, mass spectrometry^10^, ion flow-tube mass spectrometry^11,12^, surface acoustic wave sensors^13^, functionalized quartz crystal microbalance technique^14^ and chemiresistors^15^. These detection techniques despite their high selectivity and sensitivity involve sophisticated measurement instrument with higher power demand and tedious process. Thus, there is a great need to develop a more facile and energy efficient, affordable and miniaturised sensing probes for VOCs detection.

Specific advantages such as electromagnetic immunity, multiplexation capacity, online monitoring and remote introduce optical fibre sensors as a real alternative in recent years^16–18^. Moreover, the intrinsic properties of optical fibres to conduct light to a remote location, their microscopic light-coupled cross-section, and biocompatibility candidate them as the best technological substrate for a simultaneous transducer for implementation of sensing and multiplexing schemes. The combination of such optical platform with nanostructures, e. g. evanescent field-based optical fibre sensors, can significantly modify the interaction between the device and the surrounding environment and supply an attractive opportunity for sensing application. In other words, this sensor takes advantage of the synergy between both the properties of nanostructure active layers as well as the ones that charactrise optical fibre, which allows a wide range of applications concerning low cost and rapid gas detection techniques. Nevertheless, the evanescent field optical fibre sensor often suffers from lower sensitivity caused by both the inherent limitation of the employed nanostructures coated on the optical fibre and the optical sensing setup that does not satisfy the rigorous detection demands of a specific analyte with fractional contents. In this regard, two main lines have to be traced in the optical fibre-based sensors roadmap: (a) providing a suitable optical fibre sensor configuration to make an efficient transducer; (b) maximizing the analyte interaction with the sensing surface by choosing a proper nanostructure for its efficient deposition on the optical fibre in accordance with the analyte. To follow the first line of optimisation, various configurations to optimise the structures of the sensing have been reported so far such as D-type, U-shaped and tapered fibres such that the tapered fibre is the more versatile one^19–22^.

Coating nanostructure on fibre for construction of sensing region is the cornerstone of the sensor configuration to develop a simple, highly sensitive and fast response sensing system. Finding and synthesis of novel and proper nanostructure with simple and straightforward routs based on accessible and affordable starting material is of great importance. Various types of metal oxide such as ZnO, TiO_2_, In_2_O_3_, SnO_2_, CuO, CdO, Fe_2_O_3_, and MoO_3_ have been employed in the fabrication of gas sensors^23,24^, while modified cation occurs by their compositing with noble metal nanoparticles e. g. palladium (Pd), silver (Ag), and gold (Au), forming noble metal/metal oxide heterostructured sensor nano-composite for further enhancement of the sensing performance^25,26^. The other versatile sensitising agent’s category is carbon-based nanomaterials e.g. fluorine, carbon and graphene quantum dot (CQD and GQD), graphene oxide (GO) and reduced graphene oxide (rGO) owing to simple synthesis approaches have great with the scale-up potential and excellent sensitivity to the majority of gases^27–31^. Regarding that graphene renders an attractive gas sensing device due to its intrinsic properties such as large sensing surface area corresponds to the atoms in sheets which supply higher interaction with gas molecules, zero rest mass of its charged carriers and the higher carrier mobility^28–30^.

Economical sensing nanostructure construction is of great importance and according to such types of sensitising agent supplied from coal, starting material as the cheapest, available, and most abundant carbon sources is highly recommended for sensor fabrication. The previous researches reported that many carbon nano-materials, including graphene QD, agglomerated GO, CQDs and agglomerated carbon nanocrystals can be found in the coal samples^32,33^. Herein, both the nitrogen-doped carbon and graphene from natural coal through some simple treatments, such as chemical oxidation and centrifugation.

On the other hand, following best selection of modifier owing to generation of new functional and reactive centres is proportional to selectivity and sensitivity enhancement. Combination of such reactive centres in cooperation with hydrogen bonding and van der Waals forces control the selecting pattern and sensing performance.

**Figure 1:**
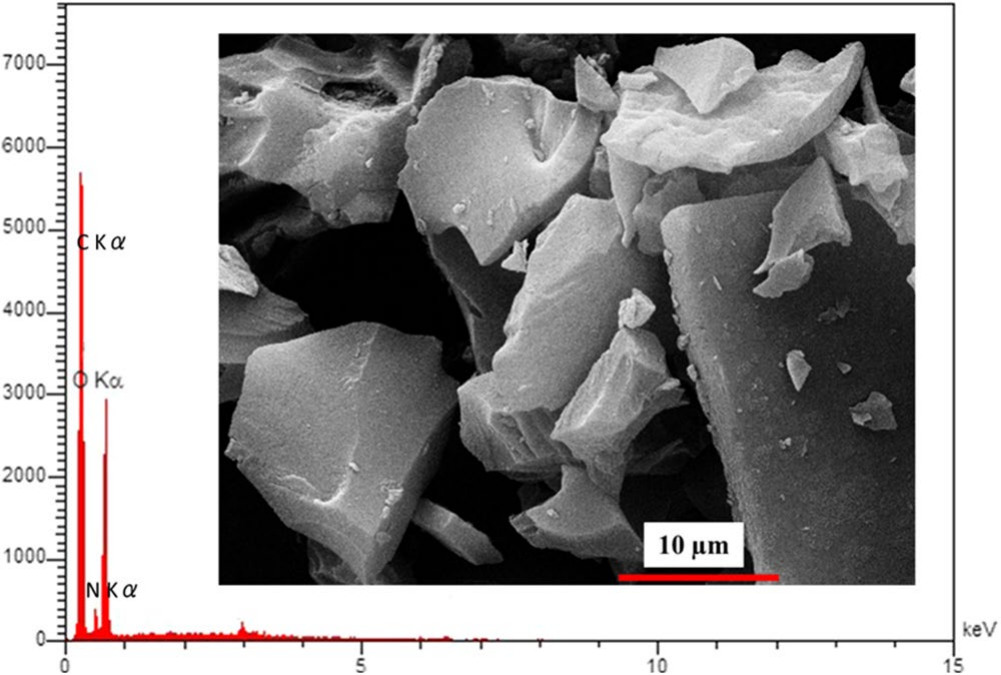
The morphology and elemental analyses of the utilised natural carbon powder.

**Figure 2:**
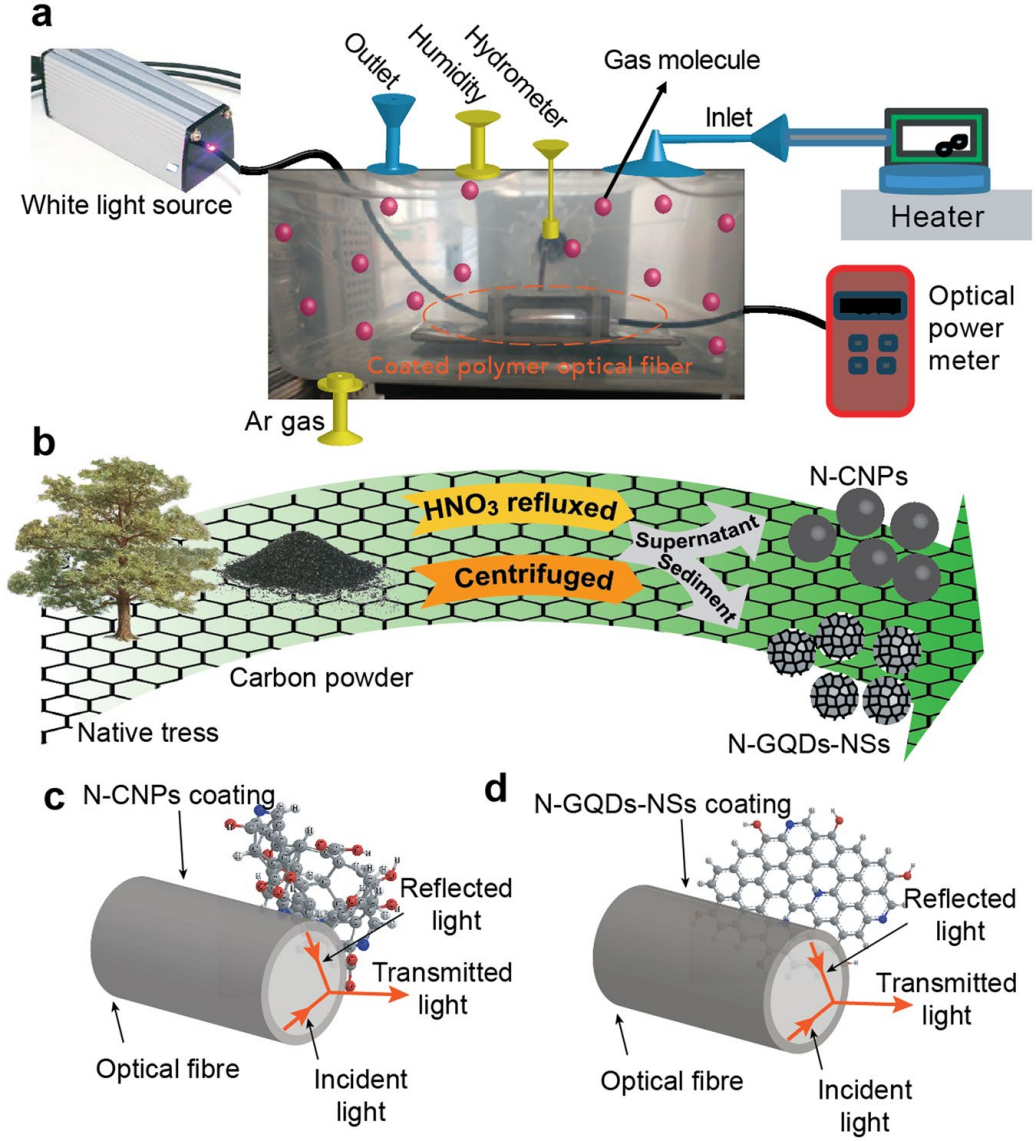
(**a**) Schematic illustration of the experimental setup used to evaluate the performance of the N-CNPs and N-GQD-NSs polymer optical fibre sensors. (**b**) Schematic present of treatment procedure and resultant supernatant and sediment as the sensitizing agents of (**c**) N-CNPs and (**d**) N-GQD-NSs overlaid onto the optical fibre.

The document is structured in the following sections: first, the sensitising agents’ synthesis and subsequently optical fibre sensor setup are presented. The next section is paying particular attention to the morphological and optical characterisation of the as-prepared sensing material. To clarify the applied treatments, the morphology and elemental analyses of the utilised natural carbon powder has been illustrated in Fig. 1. At the last section, the sensing performances of nanostructure coated optical fibre was investigated under exposure to some volatile chemical vapours in the presences of various ratio of humidity. The comparative behaviour of sensing performances of carbon and graphene-coated on the polymer optical fibre sensors implies that due to the unique properties of graphene combined with the advantages of optical fibre sensing schemes rendering it ideal for various chemical gas sensor applications. An illustration of the applied natural carbon and its treatment as sensitising agents for coating optical fibre was shown in Fig. 2.

## Results and Discussion

**Figure 3:**
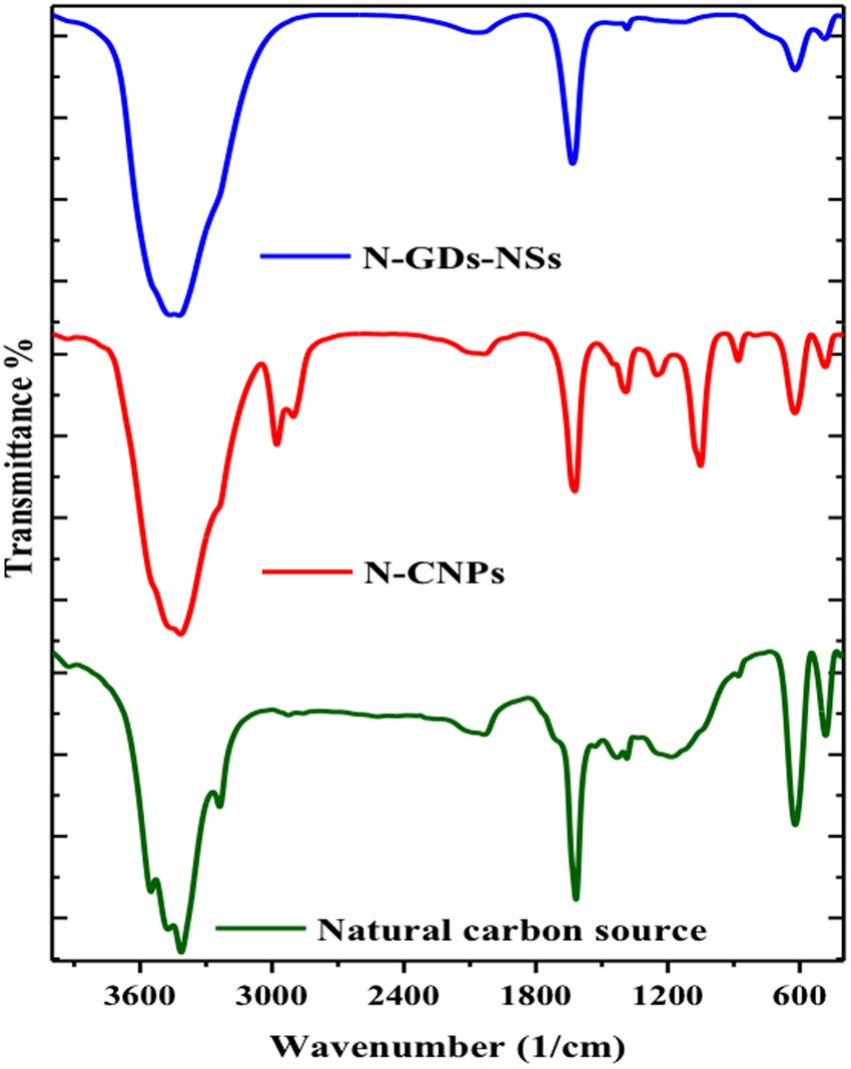
FTIR spectra obtained for N-CNPs (pink line) and N-GQD-NSs (cyan line).

The Fourier transform infrared (FT-IR) spectra show the presence of different types of oxygen and nitrogen functional groups including carbonyl, hydroxyl and epoxy, imine and nitro groups in the prepared samples (Fig. 3). FT-IR spectra of prepared samples display the prominent characteristic peaks at about 3411 cm^−1^ associated with the adsorbed water or the hydroxyl groups. In all spectra, the sub-peaks located at 3400 cm^−1^ are ascribed to the N-H and N-H_2_ bonds, vibration which clearly indicated the successful passivation of nitrogen atoms on the precursor surface^34^. The strong bands at about 1620 cm^−1^ show characteristic of the in-plane vibrations of the aromatic C=C with sp^2^ hybridised carbon in all samples. This peak is more remarkable in N-GQDs-NSs sample implying aromatic ring as a graphene structure. At the same time, the obvious broad peak extended from 1000 cm^−1^ to 1400 cm^−1^ for natural carbon which is converted to the peaks centered at 1390 cm^−1^, 1244 cm^−1^ and a notable peak of 1050 cm^−1^ for N-CNPs implying the presence of C-N, C-O-C and C-O groups, respectively. While, they were disappeared in N-GQDs-NSs structure in consistence with the XPS results displaying less oxo-and nitro-groups. These –COOH and -OH functional groups in N-CNPs structure enhance their hydrophilicity and stability of both specimens in aqueous solution. Moreover, the stretching vibrations of -CH_2_ and –CH were located around 880 cm^−1^ and 2978 cm^−1^, respectively, demonstrating that the alkyl chains became more densely packed around the surface which cannot be observed for N-GQDs-NSs^34^.

**Figure 4:**
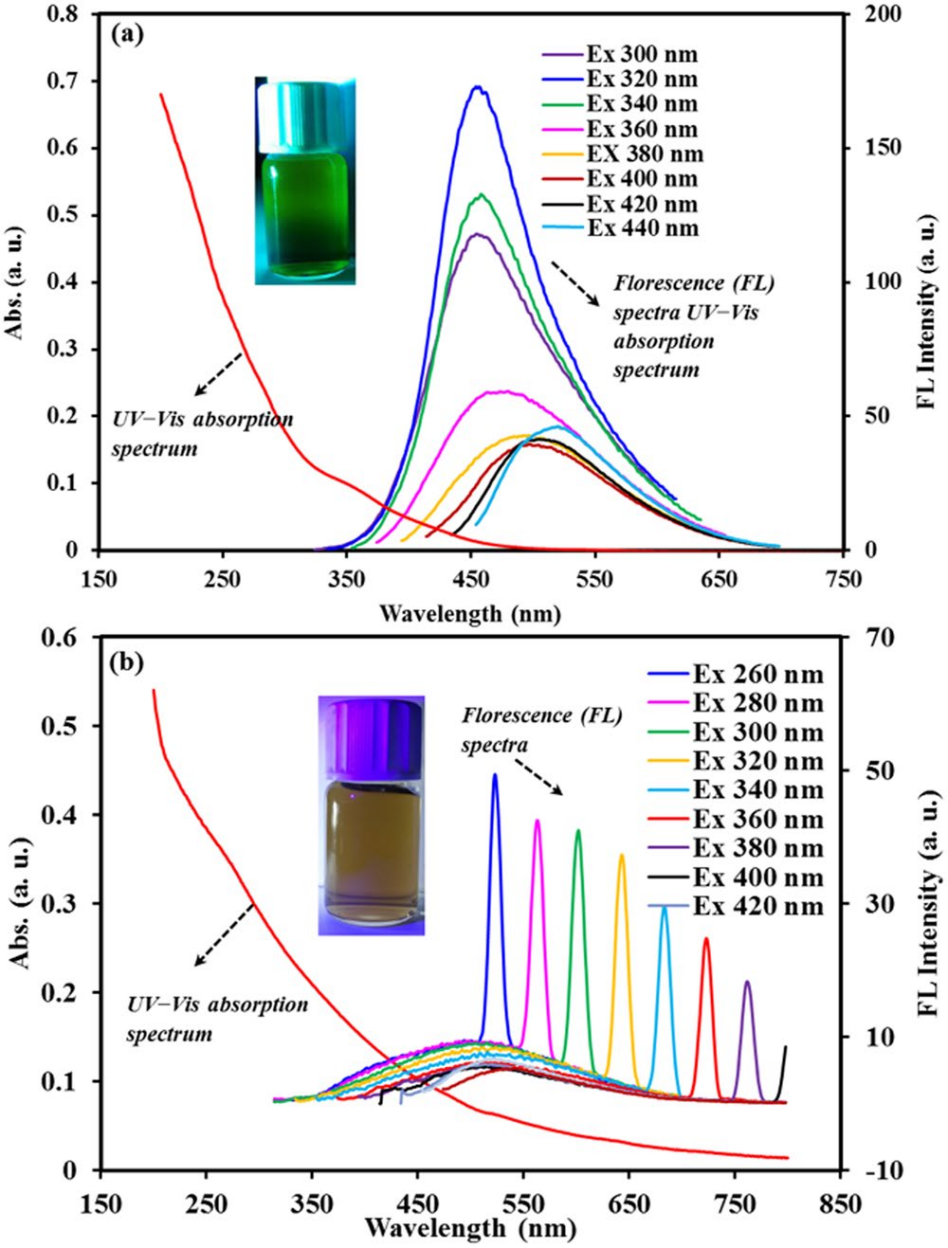
UV-vis absorption spectra and FL spectra of the aqueous solution of N-CNPs (**a**) and N-GQD-NSs (**b**) under excitation of different wavelength lights (inset: photograph for the corresponding solution excited by 275 nm).

The optical properties of the as-prepared samples were assessed by UV−Vis absorption (the continuous decaying red curves illustrated in the primary axis in Fig. 4a,b) and fluorescence (FL) spectra (spectra related to the secondary axis in Fig. 3). The UV−Vis absorption spectrum of the aqueous dispersion of N doped-CNPs (See Fig. 4a) exhibit an approximately broad absorption region from 200 to 500 nm with a typical absorption weak peak at 360 nm ascribed to the n-π* transitions of C-N owing to the presence of N-doped CNPs^35^. In addition, for N-GQDs-CNs sample, absorption spectrum (See Fig. 4b) expands to the whole-visible region with absorption edge near 640 nm. It is clearly noticed that N-GQDs-NSs do not exhibit any prominent absorption peak, which implies that N-doping or other oxygen-based functional groups do not an impressive effect on the UV-Vis adsorption process. In the inset of these figures, the photographs produced under ordinary UV light suggest that aqueous dispersion of samples are green emitting and no emitting for N-CNPs and N-GQDs-NSs, respectively.

As-synthesized N-CNPs shows fluorescence spectra with a dependence on excitation wavelength which indicates the possible surface defect of FL process^35^. When the excitation wavelength changed from 320 to 480 nm, the FL peaks of the N-CNPs exhibited a redshift from 458 to 528 nm (Fig. 4a). Excitation of N-CNPs at 320 nm indicates the highest FL intensity at 425 nm which could be attributed to multiple factors including their shape, synthesis route, conditions and surface functionalities. The FL intensity of the N-GQDs-NSs under the same conditions (Fig. 4b), is composed of weak broad FL spectra combined with some sharp peaks. It is of impressive to note that the obtained sharp peaks in Fig. 4b are related to the second harmonic generation of the spectrofluorometer corresponding to the excited wavelength. Since the emitted FL peaks of N-GQDs-NSs were weak, these harmonics emitted by the measuring instrument became observable and noticeable. The weak FL intensity emitted by samples is attributed to this fact that the obtained N-GQDs-NSs is mainly with sheet shapes (as explained in the HR-TEM results), displaying metallic characters^36^.

**Figure 5:**
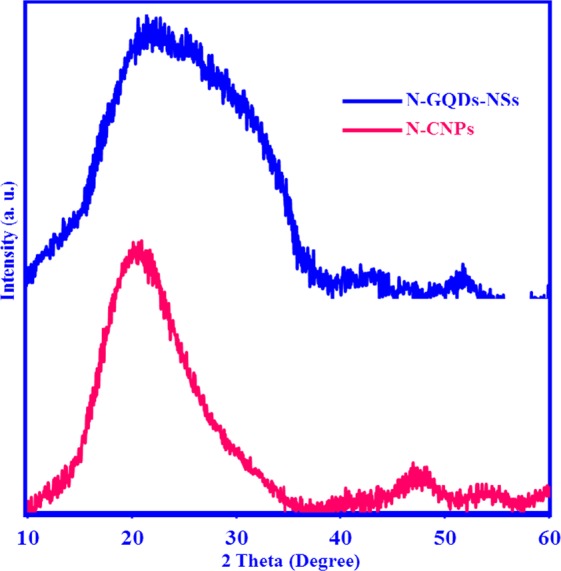
XRD patterns of N-CNPs (pink line) and N-GQD-NSs (cyan line) 2θ 10–60°.

X-ray diffraction (XRD) patterns of N-CNPs and N-GQDs-NSs (Fig. 5 red line and blue line, respectively) are composed of a broad (002) peak with centre at 2θ = 23.7 for N-GQDs-NSs and a relatively sharp (002) peak at 2θ = 20.75 for N-CNPs. The lower degree of N-CNPs indicates their most significant interlayer spacing concerning N-GQDs-NSs, which may relate to their higher oxygen and nitrogen content. Generally, carbon nanomaterials have a predominantly graphitic structure with an interlayer spacing higher than graphite^37^.

**Figure 6:**
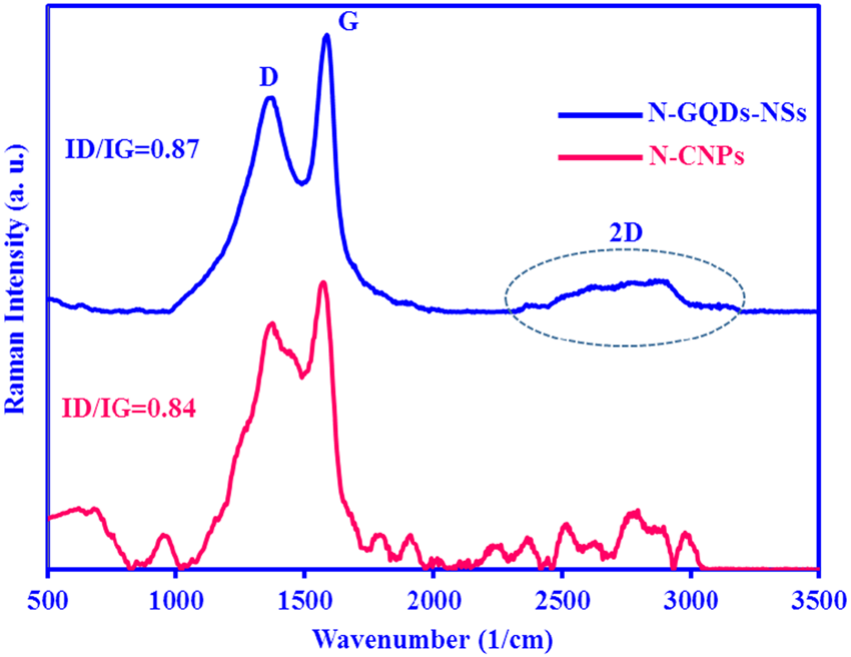
Raman spectra of N-CNPs (pink line) and N-GQD-NSs (cyan line).

The Raman spectroscopy assign to N-CNPs and N-GQDs-NSs (Fig. 6) are composed of two apparent bands located around 1590 cm^−1^ and 1383 cm^−1^, which were generally assigned as the D band (at about 1350 cm^−1^) and G band (at about 1590 cm^−1^), corresponding to the structural defects and vibration of sp2-hybridized graphitic domains, respectively. The intensity ratio of the two bands (ID/IG) is the odd indication for confirming their graphitisation degree and accordingly, lower value denotes denote a higher degree of graphitisation^38^. The ratio of ID/IG of N-CNPs was 0.84, which was few smaller than that of N-GQDs-NSs (ID/IG = 0.87). In addition, the appearance of a broad peak in the range of 2500–2800 cm^−1^ in the Raman spectrum of N-GQDs-NSs is corresponding to the vibration of sp^2^ carbon materials and graphene crystalline structure^39^. Combined with the G-band, this spectrum is a Raman signature of graphitic sp^2^ materials and is known as 2D-band that is a second-order two-phonon process and exhibits a strong frequency dependence on the excitation laser energy. The 2D band can be used to determine the number of layer of samples. This is mainly due to multi-layer samples and shape of 2D band is pretty much different from single-layer samples.

**Figure 7:**
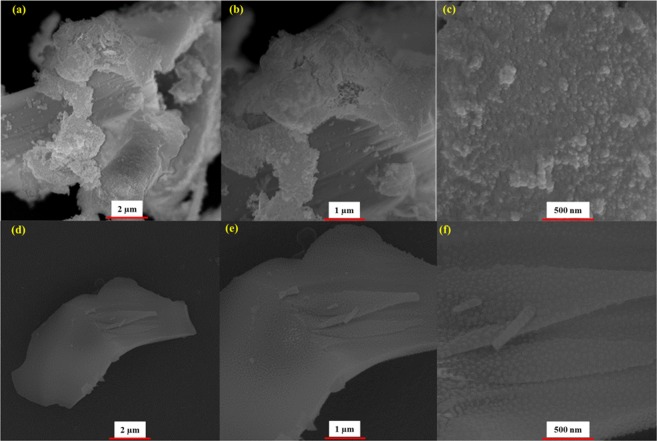
FE-SEM images of N-CNPs (**a**,**b** and **c**) N-GQD-NSs (**d**,**e** and **f**) products with scale bars of 2 µm, 1 µm and 500 nm, respectively.

**Figure 8:**
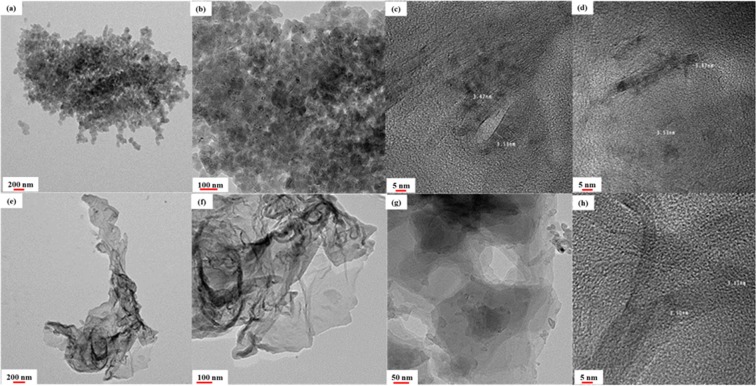
HR-TEM images of the synthesized of N-CNPs (**a**–**d**) with scale bars of 200 nm, 100 nm, 5 nm and 5 nm, respectively) N-GQD-NSs samples (**e**–**h**); with scale bars of 200 nm, 100 nm, 100 nm and 5 nm, respectively).

The morphology of the as-prepared specimens was investigated using Field Emission-Scanning Electron Microscopy (FE-SEM), High-resolution transmission electron microscopy (HR-TEM) and Atomic force microscope (AFM FE-SEM images of the N-CNPs and N-GQDs-NSs were illustrated at different magnification in Fig. 7a–f, respectively. The FE-SEM images of N-CNPs shows that the synthesised nitrogen-doped carbon particles were ultra-small with the laterally average particle size of 50 nm. The images also indicate that the samples nearly had spherical morphologies of nanoparticles with a narrow range and somewhat agglomerated distribution. As shown in Fig. 8d–f, FE-SEM images of N-GQDs-NSs show a more crystalised layers’ structure decorated with spherical nanoparticle with size less than 50 nm and nearly uniform distribution.

HR-TEM results of N-CNPs sample with different magnifications (Fig. 8a–d) show a considerable agglomeration of inordinate nitrogen-doped carbon crystals. This “disorder” structure may be attributed to the existence of the residual oxygen functional groups and the defects arising from the introduced nitrogen with more than allowed content. These crystals illustrate a lattice spacing of nearly 0.353 nm such that can be associated to the (002) facet in the graphite-like structure of this sample^40^. As shown in Fig. 8e–h, the graphene quantum dots were incorporated into graphene NSs sample by ultrasonicated treatment. In fact, the transparency observed in some segments in these figures implies few layered structure and well dispersion of graphene layers or sheets^41^. In some other segments (Fig. 8e–h) the sample shows obvious quantum dot coupled and wrinkled structure with some compact folds that imply aggregations of graphene layers probably due to the introduced nitrogen in graphene lattice and/or due to the functional groups. The obtained graphene has crystal layers with the lattice parameter of 0.35, which corresponds to the (002) fringes of graphene^42^.

**Figure 9:**
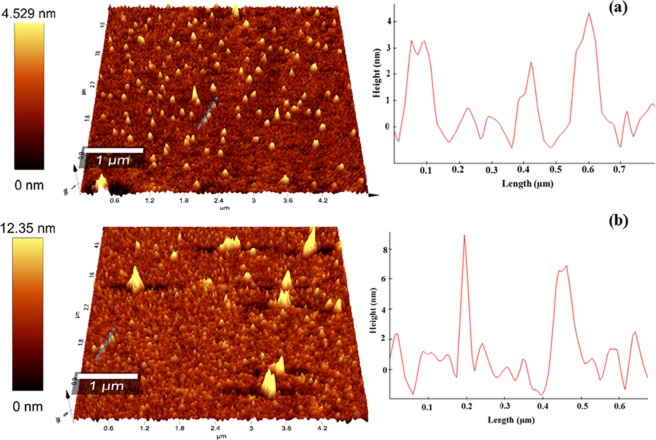
AFM topography image of nanoparticles that drop-casted from dilute solutions onto the mica substrates coated with the scale of 5 × 5 μm of N-CNPs (**a**) and N-GQD-NSs (**b**) samples and the corresponding height diagrams.

The agglomeration tendency refused by preparation of diluted solution in n-propanol, which coated on a glass slide prior to AFM investigation. The AFM image (Fig. 9a,b) reveals the existence of both small particles in the range of 20–50 nm, while smaller species being discernable as well. Both samples exhibit a high degree of uniformity in both size and shape as determined by both HR-TEM and AFM. Self-assembling with the height of lower than 8.0 nm was achieved for both samples, while N-GQDs-NSs has the size smaller than 4.0 nm with relatively smoother surface profile.

**Figure 10:**
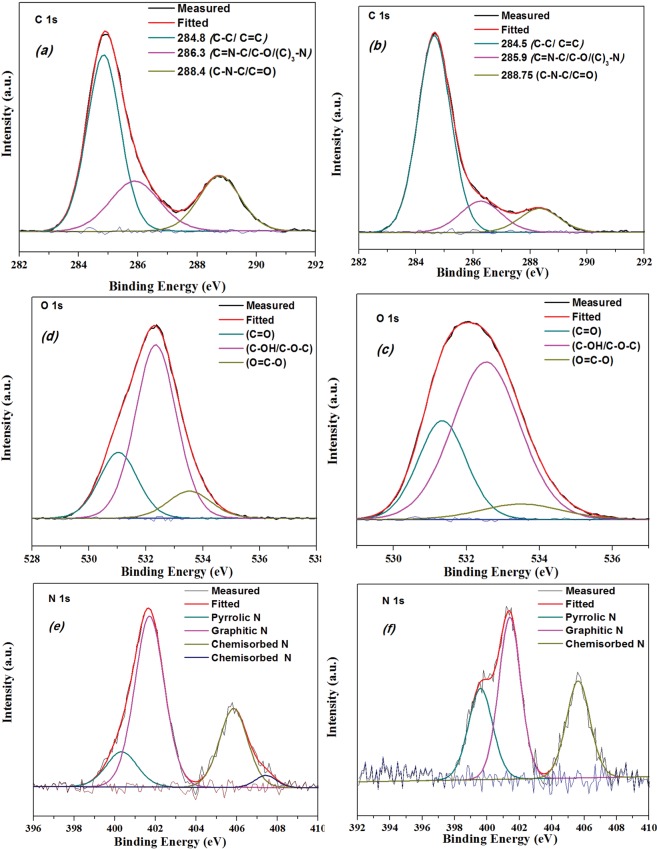
C1s, O1s and N1s XPS spectra of N-CNPs (**a**–**c**,**e**) and N-GQD-NSs (**b**–**d**,**f**).

X-ray photoelectron spectroscopy (XPS) measurements were performed on the as-prepared samples to determine their chemical information in terms of both the elemental composition and the functional groups. Herein, the surface was treated to promote the functionality of carbon materials by wet chemical treatment of boiling in nitric acid. Thus, the XPS broad scan shows the presence of significant amount of carbon and oxygen elements and small nitrogen content in the as-prepared oxidised carbon-based samples (Table 1), which are as it was expected. The obtained spectra demonstrate that for N-CNPs (N-GQD-NSs) as-synthesized samples, the C1s signal splits into three central peaks at nearly 284.85 (284.5), 286.3 (285.9) and 288.4 (288.75) eV by curve fitting spectra. The fitted curve matched with the measured results is the integrated envelope with three fitted Gaussian lines. These deconvoluted peaks are corresponding to three different types of carbon bonds e. g. graphitic and aliphatic (C–C/C=C), oxygenated and nitrous carbon. The main peak at 284.8 eV referring the sp^2^ carbon atoms displays a strong signal with the maximum fraction of 56.46% and 62.46% for the first and second samples, (Table 1) respectively. This result revealed that most of the carbon atoms are arranged in a honeycomb arrangement lattice. The other peaks of 286.3 eV, 285.9 and 288.4 eV are allocated to the sp^3^carbon of C-O, C-N and the C=O in carboxyl groups, respectively. The deconvolution of the O1s peak indicates the presence of subpeaks at around 531.2, 532.5 and 533.5 eV as shown in Fig. 10, which could be related to the existence of oxygen containing functional groups such as C=O, C-OH/C-O-C and O=C-O, respectively^43^. It can be concluded that different carboxyl and hydroxyl groups functionalized the surface of samples. To elucidate nitrogen configurations, the N1s peak was studied by splitting into the three individual peaks for the first (second) sample located at 400.34 (399.6), 401.7 (401.4) and 405.84 (405.6) eV that were labelled as NP1, NP2 and NP3 for every individual sample. These N1s spectra confirm that nitrogen was successfully introduced into the as-prepared carbon-based frameworks^39^. NP1 represents the cyanide N with a triple bond between a nitrogen and a carbon atom (N≡C), or the Pyrrolic N where one nitrogen atom is connected with two carbon atoms and one hydrogen atom (H-N-C2) in a five-membered ring. NP2 represents the graphitic N (or quaternary N) where one N atom connected to three sp^2^- hybridized carbon neighbuors in the hexagonal ring. NP3 could be attributed to both the pyridinic-N oxide, which forms direct bonds with one oxygen atom and the chemisorbed nitrogen.

**Table 1:** Elemental analysis of as-prepared samples.

	C (%)	N (%)	O (%)
N-CNPs	56.46	5.92	38.25
N-GQD-NSs	62.49	4.59	33.45
	**Pyrrolic N (%) & Peak wavelength (nm)**	**Graphitic N (%) & Peak wavelength (nm)**	**Chemisorbed N (%) & Peak wavelength (nm)**
N-CNPs	0.67 & 400.3	3.1 & 401.7	1.52 & 405.8
N-GQD-NSs	1.11 & 399.6	1.8 & 401.4	1.16 & 405.63

The graphitic N components were found to be the most prevalent which correspond to nearly 50% of the total nitrogen content and the chemisorbed N components are roughly similar with 28% of total nitrogen for both samples (in Table 1). The pronounced difference between the as-synthesized carbon-based samples are observed in the appeared variation in Pyrrolic N peak, which is shifted towards the lower peak position from 400.34 to 399.6 eV with the higher surface atomic compositions ratio from 0.67% to 1.11% from the first to the second sample. It might be attributed to this fact that some of the triplet carbon bonded nitrogen atoms replaced by the Amine-N^44^. Additionally, the ratio total surface N content in the first sample lead to efficiency around 5.29% which is higher than that in the second sample, 4.06%; similarly, the oxygen content (38.25%) was strongly diminished to 33.45% from former to the latter sample (Table 1). Graphitic N presents a graphite with divacancy defects and a marginal influence on the graphene structure due to the possessing of the nearly similar bond length of 1.41°A^45^.

Moreover, Pyrrolic N with sp^3^ hybridized bond also disrupts the graphene’s honeycomb structure. Consequently, the existence of nitrogen atoms into the structure is accompanied with the structure defects such as bonding disorders and vacancies. Thus, the nitrogen-doping would considerably influence the structure and it is a limited value possible achievement of graphene lattice^46^.

In order to arrange the obtained results based on the performed analyses and to make a conclusion, we have summarized the main points so that they can guide us to characterize and recognize properly the samples. For the second sample; firstly, the appearance of a broad peak in the range of 2500–2800 cm^−1^ in the Raman spectrum of N-GQDs-NSs assigns the crystal structure with the sp^2^ carbon atoms. Secondly, the transparent layers in TEM images for the second sample, Fig. 8e–h, imply the sheets which were coupled and wrinkled structure with some compact folds. These results support the completely different FL spectra achieved for each samples such that they indicate green emitting and no emitting for first and second samples, respectively. Finally, in order to clarify the carbon bonding nature and to confirm our previous analyses, we also considered the XPS analyses, as well. The strongest peak in the second sample (labelled as N-GQD-NSs) centered at 284.6 eV with 62.46% relative intensity. In the literature, 284.7 ± 0.2 component is attributed to sp^2^ hybridization of C-C or C-H bonds^47^. In the C1s spectrum of N-GQD-NSs (Fig. 10,b), the ratio of this peak to the other two peaks is higher than that of occurred in the first sample (labelled as N-CNPs). The dominance of sp^2^ carbon bonding confirms the graphene like structure. Accordingly, for the first sample, the denotation of carbon nanoparticle according to the presented analyses would be clear. Therefore, the overall obtained results reveal that the prepared samples have different carbon phases of amorphous carbon nanoparticles for the first sample and graphene sheets coupled with quantum dot for the second sample.

**Figure 11:**
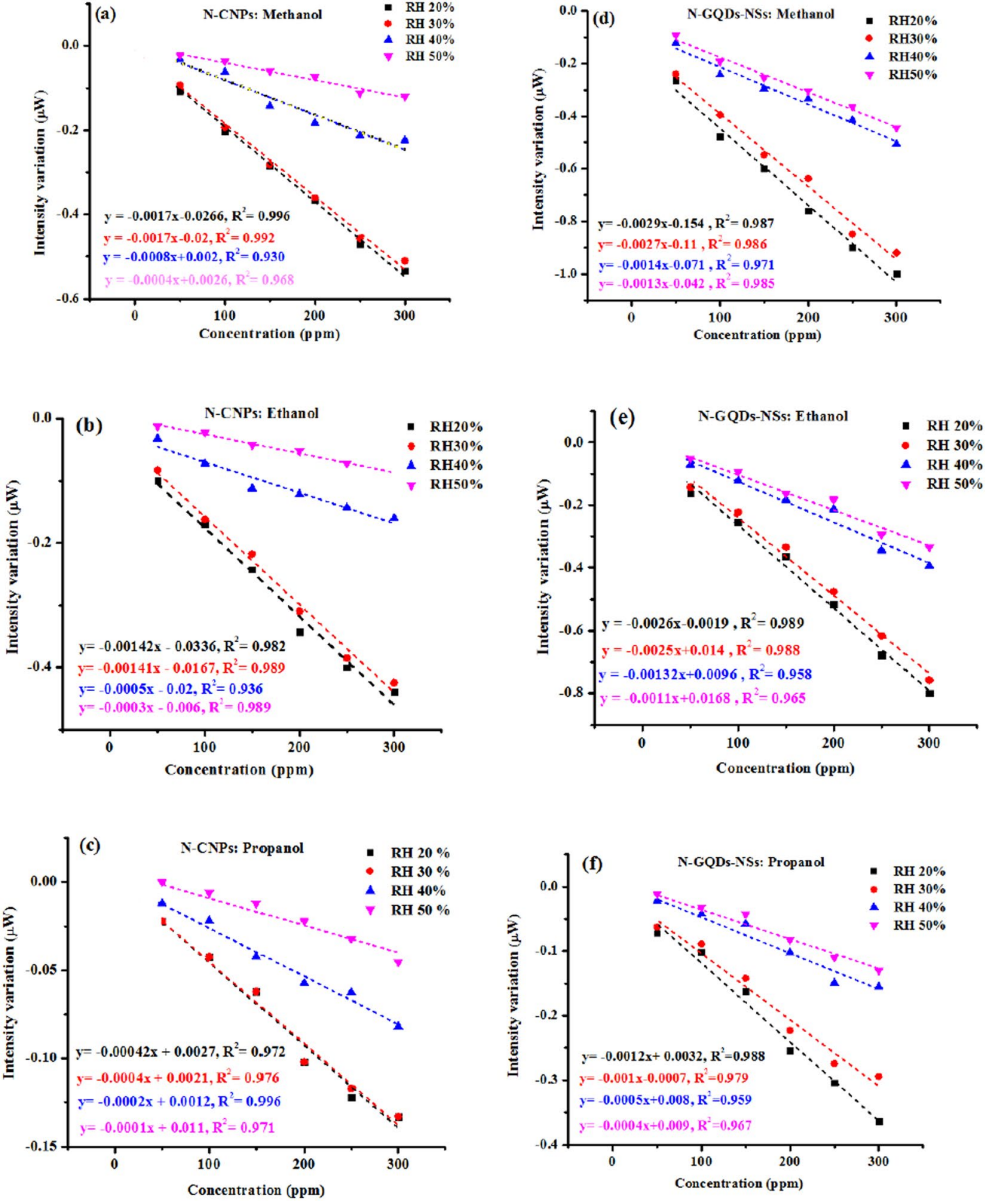
Output intensity variations of N-CNPs (**a**–**c**) and N-GQD-NSs (**d**–**f**) coated polymer optical fibre sensors as a function of the methanol, ethanol and propanol concentration, respectively in presence of different relative humidity.

The polished surface of the optical fibre was cleaned and coated with as-prepared CNPs and graphene by drop- casting technique and finally dried in air. A cubic flask was employed as a chamber in which the fibre optic sensor was inserted (Fig. 2). This enclosed test chamber is contains three inlets for entrance of humidity, Argon and alcoholic vapours. During the sensing operations, Ar gas was employed as the carrier gas and diluting gas to remove the analyte from chamber following measurement. Initially, Ar gas was passed through the test chamber to achieve a steady-state condition. VOC produced by vapourizing alcoholic solution (in a small vial on the heater as shown in Fig. 2) was directly passed into the gas chamber through the gas inlet without any carrier gas. As shown in Fig. 2 to facilitate this study, nanostructure-overlaid optical fibres were positioned in a holder inside gas sensing chamber that allowed the whole surface of fibre to be uniformly exposure to the ambient environment variations. Precise measurements without surrounding light interferences were achieved under the following condition of the dark room at normal atmospheric pressure conditions and room temperature. The sensor response was recorded for each vapour’s concentration in a various ratio of humidity. The sensing performance under various relative humidity was studied and compared by following insertion of a hygrometer in the chamber. Same sensing measurement procedure was adapted for detecting the different concentrations (from 50 to 300 ppm with 50 ppm step) of methanol, ethanol and propanol chemical vapours. Figure 11 shows the comparative results of the both N-CNPs and N-GQD-NSs coated fibre sensors of the output intensity variation against the mentioned vapours concentration. The slope of calibration curves, the intensity variation to the injected vapours concentration with the unit of µW/ppm, was calculated for all cases referring the sensitivity and the characteristic performance for each species are listed in Table 2. Results strongly support that the both N-CNPs and N-GQD-NSs coated fibres show different sensitivities depend on the type of vapour structures. The response of proposed optical fibre-based sensors as the output light intensity of the unclad fibre which was coated with suitable nanostructure undergoes an appreciable attenuation by introducing vapours into the chamber. The optical losses could be attributed to evanescent wave absorption in the modified cladding and were described in detail in our previous works by the application of the Beer-Lambert law^48^. Accordingly, the refractive index of fibre cladding material changes and lead to changing the propagating light intensity. Highest response was seen for methanol vapours, and lowest value achieved for propanol vapours at the same concentration due to the less hydrogen bonding power and its larger size corresponds to propanol.

**Table 2:** The performance of the proposed VOC detection sensors.

Gas	Sample	Sensitivity (µW/ppm)	Linearity	LOD(ppm)
Methanol	N-CNPs	−0.0017	99.7%	7.5
	N-GQD-NSs	−0.0029	99%	4.3
Ethanol	N-CNPs	−0.0014	99.2%	9.2
	N-GQD-NSs	−0.0026	99.6%	4.9
Propanol	N-CNPs	−0.0004	99.1%	32.25
	N-GQD-NSs	−0.0012	99.3%	10.48

The limit of detections (LOD) at 95% confidence interval based on the standard deviation of the response and the slope of calibration curve were calculated according to this definition^49,50^

1 $$LOD=\frac{3{S}_{Blank}}{m}$$

where denotes the standard deviation of responses for blank and m is the slope of a linear calibration line. This value was calculated for both sensors and summarized in Table 2.

The relatively low value of the present sensor in comparison to the reported similar works^51–53^ is a good indication for its suitability for analyte monitoring in complex media at trace level. The lower values for N-GQD-NSs based sensor is owing to the higher sensitivity of this material.

**Figure 12:**
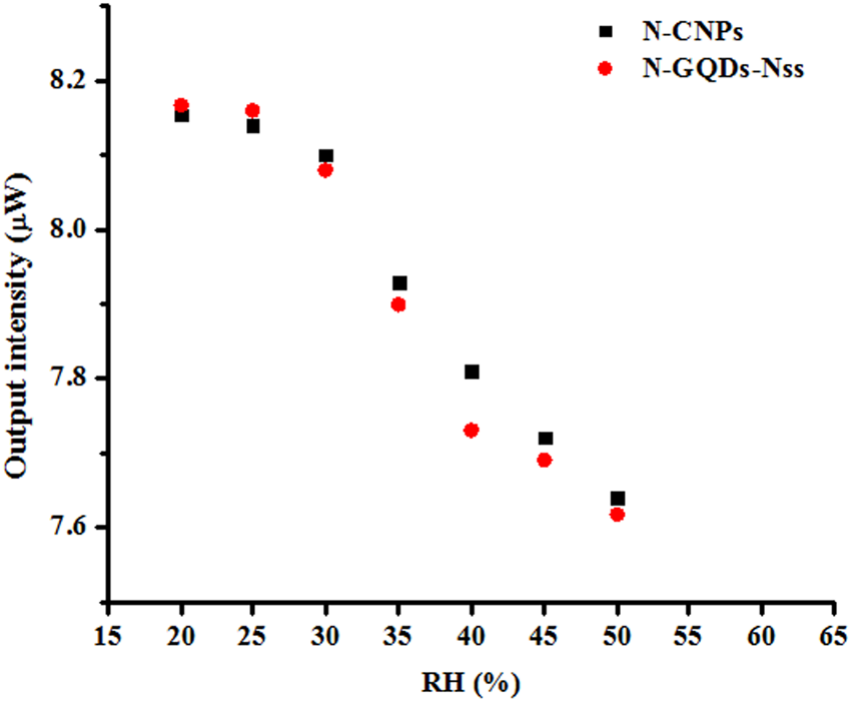
Output intensity of N-CNPs and N-GQD-NSs coated polymer optical fibre sensors against the relative humidity at a constant concentration of 100 ppm of methanol vapour.

To further evaluation of the proposed sensors performance, identical investigation procedure was followed under dry air and humid air environment which implemented by exposing the different relative humidity percentages (20, 30, 40 and 50%). The results for every individual new condition were indicated in Fig. 12. It is evident that the lower water vapour in the surrounding is associated with the highest sensitivity as shown in Fig. 12. For instance, the sensitivity of carbon coated fibre decreases in exposure to methanol from −0.0017 µW/ppm to −0.0008 µW/ppm at 20 to 40% relative humidity. Accordingly, the sensors show the same sensing behaviour for 20 and 30% relative humidity, while sensitivity significantly decreased at 40%. The measured output responses show quite weak dependence on the humidity variations between 20 and 30% relative humidity concentrations. This result suggests that the sensors may be close to humidity saturation at the ranges of 40 or 50%. Therefore, the proposed sensor might be more useful in the arid regions.

**Figure 13:**
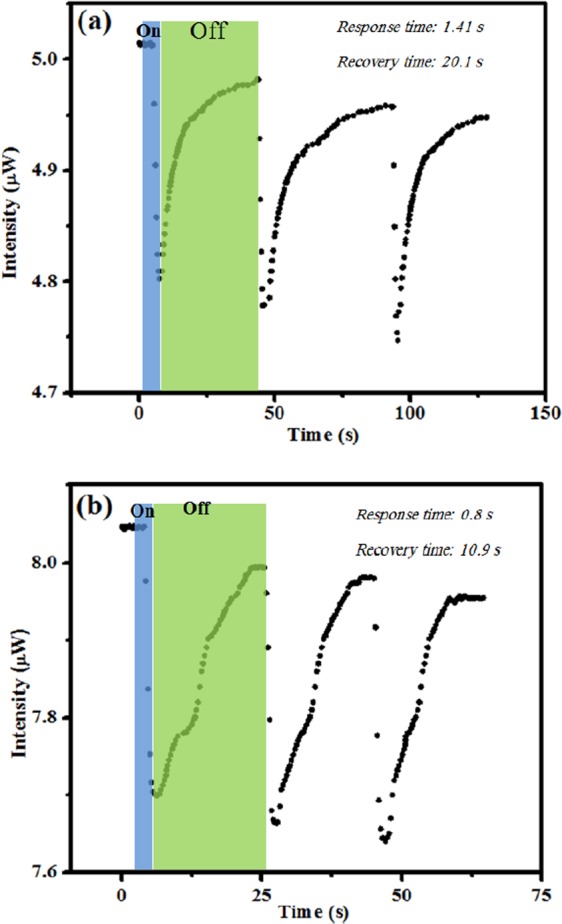
Time response of N-CNPs (**a**) and N-GQD-NSs (**b**) coated polymer optical fibre sensors for 100 ppm ethanol vapour at room temperature.

Referring to the demonstrated results (Fig. 13(a,b)) both sensors immediately respond to the vapours. The estimated response and recovery time is a few and a few tens of seconds, respectively. Achievement of steady-state requires that the outlet was opened and sample diluted exposing the sample to Ar gas to fully recovered back to its initial intensity. This cycle was repeated for a number of times and the sensor response was recorded according to the exposing sensor based on both N-CNPs and N-GQDs-NSs coated fibres to the methanol vapour with 100 ppm concentration in the chamber as illustrated in Fig. 2. The relative standard deviations (RSD) of 1.29% and 2.39% were calculated for carbon and graphene-coated fibre sensors, respectively in a constant atmosphere condition. The results that are shown in Fig. 13 and the obtained RSD values of 2.4% and 1.3% for N-CNPs and N-GQDs-NSs optical fibre based sensors, respectively confirm the repeatability of the proposed sensing probes for successive cycles under ambient conditions and at room temperature. Generally, the proposed N-CNPs and N-GQDs-NSs coated optical fibre sensors exhibited a well-established high sensitivity and a noticeable linear response over the concentration range of 50 to 300 ppm. Sensing performance of the proposed sensors provide an overview of fast response and recovery time of the device compare to reported literatures as illustrated in Table 3. These remarkable mentioned improvements in the sensing performance could be owing to the exploiting firstly polymer optical fibre as a platform and secondly the coating of the fibre with N-CNPs and/or N-GQDs-NSs as sensitising agents. The reasons behind this fact were explained in more details as follow. In polymer fibres, light propagates through the fibre with producing more modes, leading to more leakages of light modes and so, more penetrated evanescent waves in the unclad region, resulted in more interaction with the surrounding ambient environment. The other factor, which plays a crucial role in the clad-modified fibre optics sensors, is the optical and chemical features of the employed nanostructure. Herein, N-GQDs-NSs coated optical fibre sensor exhibited more impressive sensing improvements rather than carbon coated one. These comparable sensitivity features of graphene in a chemical environment can be due to several reasons: First, graphene possesses extremely high electron mobility and high electrical conductivity. Second, every carbon atom in graphene is a surface atom such that it presents the greatest possible surface area per unit volume, leading to the highly sensitive surface to the adsorbed gas molecular species. Third, graphene exhibited a broad absorption band within UV-Vis spectral region, which is extended its application in optical-based devices^54^. These versatile properties make graphene an ideal candidate for the ultrahigh sensitivity detection of different VOC gases in various environments.

**Table 3:** Comparison of VOCs sensor response and recovery time employing coated optical fibres. SMF, FBG, PMMA, SWCNT, and MWCNT are the abbreviation for single mode fibre, fibre Bragg grating, Polymethyle methacrylate, single-walled carbon nanotube and multiwall carbon nanotube, respectively.

Optical fibre	Analyte	coating	Response /Recovery time	Ranges	References
SMF	Ethanol	SnO_2_	8 s/64 s	1000–20000 ppm	^60^
FBG	Ethanol	PMMA	52 min/78 min	0–3%	^61^
PMMA	Ethanol	SWCNT	90 min/60 min	0–500 ppm	^62^
POF	Acetone	GO	2 min/1.5 min	500ppb-500ppm	^43^
POF	Ethanol	Novolac-resin	20 s/80 s	30–90 mm Hg	^63^
PMMA	Ethanol	GO-MWCNT	<60 min/<70 min	0–500 ppm	^64^
POF	Ethanol	N-GQD-NSs	0.8 s/10.9 s	50–300 ppm	This work

## Conclusion

In conclusion, we have presented a very simple and facile method for synthesizing nitrogen doped-carbon nanoparticle and graphene quantum dots coupled with nanosheets from natural carbon powder. The proposed strategy is not only easier and cheaper than the usual methods because of the cheaper starting material; it can also make two main products of nitrogen doping-carbon and graphene functional materials by extending a simple preparing technique. The doping N atoms consist of graphitic, Pyrrolic and chemisorbed with total surface atomic compositions of nearly 5.92 and 4.59% were obtained in N-CNPs and N-GQDs-NSs, respectively. Moreover, the sensing features of the prepared samples were experimentally studied in conjunction with a polymer optical fibre in a compact and simple intensity-based sensing setup for methanol, ethanol and propanol vapours detections. The as-prepared N-GQDs-NSs exhibited an enhanced sensing performance with an average sensitivity of around −0.002 µW/ppm and a linearity of more than 98% at room temperature. Overall results revealed the as-synthesized agents coated the polymer optical fibre could be a promising candidate for gas sensing applications at room temperature with relative humidity presence up to 30%.

## Materials and Methods

### Materials and apparatus

The starting material, coal, is produced by the incomplete combustion of natural wood (*Yasouj city’s trees*). The Energy-dispersive X-ray spectroscopy (EDS) spectrum of the used natural carbon powder confirms the presence of carbon, nitrogen and oxygen (as shown in Fig. 1). As it can be observed, its morphology is randomized with large rock-shaped sizes. This coal was applied to the preparation of carbon and graphene-based nanomaterials. Other chemicals for this work were purchased from Merck Company and used without additional purification. The absorbance of solutions was measured using a UV–Vis spectrophotometer (model PG 180 + instrument, England). Fluorescence spectra (FL) were obtained using a QM40 fluorescence spectrophotometer (PTI Ltd, Canada). The surface morphology of the as-prepared specimens was studied by a scanning electron microscope (TESCAN S8000 FE-SEM, USA). The morphology of samples was further examined with Transmission electron microscopy (TEM). To perform TEM, samples were dispersed in ethanol, and the few drops of this solution were drop casting onto the carbon grids. Then, the grids were dried in air at room temperature. The casting over the grid was such that it was able to support the image process. Infrared (FTIR) spectra were recorded in the 400–4000 cm^*−*1^ range with Perkin-Elmer spectrometer according to the KBr pellet technique by mixing about 1 mg of the sample and 300 mg of KBr. X-ray photoelectron spectroscopy (XPS) analysis was done on the Thermo Fisher Scientific K-Alpha 1063 X-ray photoelectron spectrometer (Thermo Fisher Scientific, Britain) using Al, K as the exciting source. An x-ray diffraction (XRD) instrument (Rigaku 2500, Japan) was employed to characterize the morphology of the as-prepared materials. Atomic force microscope (AFM) image of samples was obtained using an Alpha 300 (WITec, Germany). The ultrasonic processor UP200S (200 watts, 24 kHz, Hielscher Ultrasonics Germany) was used for sonication during the synthesis of sample.

### Preparation of sensitizing agents

In this simple and facile method, 0.8 g of coal powder was refluxed with 200 mL of 6 mol/L HNO_3_ at 90 °C for 48 h and subsequently the resultant suspension was cooled to room temperature and followed by three times centrifuging for 15 min at 4000 rpm was undertaken for phase separation. The centrifuged resultant, which includes supernatant and sediment, undergoes two different sensitising materials (Fig. 2). The supernatant namely as nitrogen-doped carbon nanoparticles was dried at 200 °C by a heater while solid sediment was washed with HCl (1 mol/L) for three times and dried in an oven. Then, it was dispersed completely in 200 ml double-distilled water and its pH was adjusted at 8 via ammonia. Finally, the dried obtained powder as nitrogen-doped graphene quantum dots coupled to nanosheets was dissolved in water under sonication (Sonics Vibracell, 1500 W, 20 kHz, 30 °C) for 1.0 h.

### Optical sensing setup

A polymer cladding multimode fibre with the numerical aperture of 0.2 ± 0.015 was used to make a sensing region substrate. Polymer optical fibre (POF) has been applied due to the possessing some especific physical and optical properties. The flexibility, large core sizes and high numerical apertures in these fibres provide the ease of the light intensity level monitoring. Accordingly, the simplicity in design associated with the efficient light coupling has resulted in their development as intensity-based sensors^55,56^. Removing the fibre’s cladding, i.e. tapering fibre, is the essential starting steps for the sensors based on the evanescent wave. Tapered optical fibre not only exposes the evanescent field to the surrounding environment, (Fig. 2b–d), but also increases the evanescent field magnitude, penetration depth and consequently, enhance the potential of the optical fibre as a sensor^57,58^. In this experiment, first the outer cladding of 1.0 cm length in the middle part of POF was removed using a homemade mechanical stripper. Following this, the obtained fibre was smoothed and polished by applying sand papers with different roughnes. The removed clad segment of the fibre was then cleaned by mixture of acetone with methanol (volume ratio of 3:1). It is worth noting that before using the sanding technique, we tried to remove fibre clad by immersing it in the HF acidic solution according to our previous works^48,59^ which were done for the multi-mode fibre, the obtained fibre became very fragile and broken easily during the sensing implementation. This process made us to apply sanding technique even though it takes much more time. Schematic diagram of the proposed setup for light intensity measurement through the clad-modified fibre is shown in Fig. 2a. A white light source (model: HNK-HP003-E45) with spectral wavelength range of 400 nm to 800 nm was irradiated into the one end of fibre. To record the transmitted light intensity of the fibre, the other end of the prepared sensor was directly coupled with an optical power meter (model: Thorlabs-PM20A). The preliminary testing of the intensity fluctuations of the used light source was applied to calibrate the output.
